# Editorial: A Comparative Survey of the RF-Amide Peptide Superfamily

**DOI:** 10.3389/fendo.2015.00120

**Published:** 2015-08-10

**Authors:** Karine Rousseau, Sylvie Dufour, Hubert Vaudry

**Affiliations:** ^1^Laboratory of Biology of Aquatic Organisms and Ecosystems (BOREA), Muséum National d’Histoire Naturelle, CNRS 7208, IRD 207, Université Pierre and Marie Curie, UCBN, Paris, France; ^2^Laboratory of Neuronal and Neuroendocrine Differentiation and Communication, INSERM U982, International Associated Laboratory Samuel de Champlain, Institute for Research and Innovation in Biomedicine (IRIB), University of Rouen, Mont-Saint-Aignan, France

**Keywords:** RF-amide peptides, receptors, evolution, functions, deuterostomes, protostomes

The first member of the RF-amide peptide superfamily to be characterized, in 1977, was the cardioexcitatory peptide, FMRFamide, isolated from the ganglia of the clam *Macrocallista nimbosa* (1). Since then, a large number of such peptides, designated after their C-terminal arginine (R) and amidated phenylalanine (F) residues, have been identified in representative species of all major phyla. The discovery, 12 years ago, that the RF-amide peptide kisspeptin, acting via GPR54, was essential for the onset of puberty and reproduction, has been a major breakthrough in reproductive physiology (2–4). It has also put in front of the spotlights RF-amide peptides and has invigorated research on this superfamily of regulatory neuropeptides. The present Research Topic aims at illustrating major advances achieved, through comparative studies in (mammalian and non-mammalian) vertebrates and invertebrates, in the knowledge of RF-amide peptides in terms of evolutionary history and physiological significance.

Since 2006, by means of phylogenetic analyses, the superfamily of RFamide peptides has been divided into five families/groups in vertebrates (5, 6): kisspeptin, 26RFa/QRFP, GnIH (including LPXRFa and RFRP), NPFF, and PrRP. Recent data reveal that SIFamide-type neuropeptides in protostomian invertebrates and SALMFamide-type neuropeptides in deuterostomian invertebrates share a common evolutionary origin with vertebrate LPXRFa and PQRFa (7). Comparative studies on non-mammalian vertebrates and invertebrates allow major advances in the knowledge of the evolutionary history of the RF-amide peptide superfamily. Such phylogenetical studies also contribute to refine classification and nomenclature of both peptides and receptors. In this issue, Yun et al. (8) show that the concept of coevolution of peptide ligands and their cognate receptors helps to re-examine not only the classification of receptors but also their peptides. They thus report that kisspeptin should be classified in the galanin/spexin family rather than in the RF-amide peptide family. Another example is given by Tachibana and Sakamoto (9) who propose non-mammalian PrRP (C)-RFa to be renamed PrRP2. With the identification of the QRFPR genes in coelacanth and spotted gar, Larhammar et al. (10) demonstrate that the QRFP system is complex in the early stages of vertebrate evolution and secondarily becomes restricted in mammals.

In their review, Elphick and Mirabeau (11) recount the occurrence of the RFamide motif in bilaterian neuropeptide families. They report that peptides, such as NPY/NPF, have acquired modified C-terminal characteristics in vertebrates, while RFamide-type peptides like luqins have been lost in the vertebrate lineage. They also underline some neuropeptide families (e.g., CCK/sulfakinins) in which the RFamide motif is unique to protostomian members. Osugi et al. (12) show that identification of GnIH in agnathans (lamprey) and amphioxus reveals that the C-terminal amide motif of GnIH can differ, being QPQRF or RPQRF, in addition to previously observed LPXRF in birds, mammals and most of fish, and MPQRF in grass puffer and medaka.

As indicated above, the characterization of the first RF-amide peptide was carried out in a mollusk. Since then, many different genes have been identified in invertebrates, and the reviews by Zatylny-Gaudin and Favrel (13) in mollusks, and by Li and Kim (14) and Peymen et al. (15) in nematodes, emphasize the need of identifying receptors for these peptides in invertebrates and characterizing their signaling pathways.

RF-amide peptides from different families have major evolutionary conserved roles in the control of reproduction, food intake, metabolism, energy expenditure, cardiovascular function, nociception, and stress (16–20). The review by Ayachi and Simonin (21) presents the emerging evidences in rodents that all RF-amide peptides and their receptors are involved in the modulation of nociception in basal and chronic pain conditions, as well as of opioid-induced hyperalgesia. The reviews on GnIH by Osugi et al. (12) and by Ogawa and Parhar (22) report that even if the inhibitory role of GnIH is well established in later-evolved vertebrates, such as birds and mammals, the situation is less clear in teleosts and may vary according to the maturational stage. Comparative studies have the potential to reveal novel regulatory mechanisms that could give a better comprehension of physiological functions. Interestingly, in invertebrates, as in vertebrates, multiple genes as well as multiple mature peptides are often present in a single species, questioning the need for such diversity in term of function. In this Research Topic, Tachibana and Sakamoto (9) report that physiological actions of PrRP and PrRP2 seem to overlap in non-mammalian vertebrates, while converging into those of PrRP in mammals. Studies on lower vertebrate models can also contribute to the discovery of new roles of these peptides. For example, Sandvik et al. (23) review the role of RF-amide peptides in development, first established in medaka (24). In addition, Bouteau et al. (25) provide the first evidence that FMRFamide like peptides (FLPs) may be involved in physiological processes related to hyperosmotic stress responses in plants, widening the scope of RFamide peptides far beyond bilaterian perspectives.

We are particularly indebted to all the researchers who have enthusiastically answered to our invitation to contribute articles to this Research Topic, and to the reviewers who helped us reach the highest standards. It is our hope that this Research Topic will become a major set of references for those working on the phylogenetic history of RFamide-related peptides, and will raise interest of others who are not (yet) involved in this research area.

## Conflict of Interest Statement

The authors declare that the research was conducted in the absence of any commercial or financial relationships that could be construed as a potential conflict of interest.
